# Electrospun Fiber Mats with Metronidazole: Design, Evaluation, and Release Kinetics

**DOI:** 10.1021/acs.jpcb.5c00873

**Published:** 2025-04-03

**Authors:** Olga Adamczyk, Aleksandra Deptuch, Tomasz R. Tarnawski, Piotr M. Zieliński, Anna Drzewicz, Ewa Juszyńska-Gałązka

**Affiliations:** † Institute of Nuclear Physics, Polish Academy of Sciences, Krakow PL-31342, Poland; ‡ Research Center for Thermal and Entropic Science, Graduate School of Science, Osaka University, Toyonaka, Osaka 560-0043, Japan

## Abstract

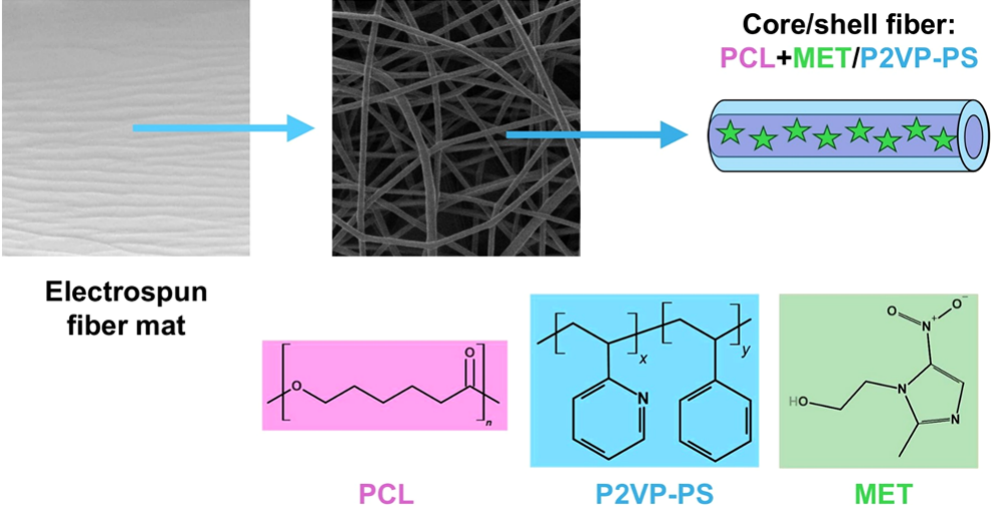

Novel drug delivery systems (DDSs) strive to eliminate or at least reduce the side effects and limitations associated with conventional medical products. Among the many potential candidates for DDSs, there are one-dimensional micro- and nanostructured materials such as electrospun fibers. In this study, two different polymers, i.e., amphiphilic block copolymer (poly(2-vinylpyridine-*co*-styrene)) and hydrophobic polymer (polycaprolactone), were utilized as base materials for fibers. Through the electrospinning and coaxial electrospinning techniques, fibers with diverse architectures were obtained, homogeneous or core/shell structures. An antibacterial drug (metronidazole) in varying concentrations was incorporated into the electrospun fibers. The potential application of the obtained electrospun fiber mats is as a dressing for wounds or the treatment of periodontitis. The average diameter of fibers fell within the range of 700–1300 nm, with a drug content of 7–27 wt %. The amorphization or decrease in crystallinity of metronidazole present in the fibers was achieved during the electrospinning process. In vitro drug release tests showed that burst effects can be successfully suppressed, and more sustained release can be accomplished for some formulations. Therefore, electrospun polymer fiber mats are promising candidates for the local delivery of active substances.

## Introduction

One of the most widely employed strategies in designing drug delivery systems (DDSs) is the nanoconfinement or encapsulation of drug molecules within various structures, such as mesoporous silica nanoparticles,^1,2^ electrospun fibers,^3,4^ metal–organic frameworks,^5,6^ carbon nanotubes,^7,8^ dendrimers,^9,10^ or liposomes.^11,12^ This approach ideally offers several benefits, including enhanced drug stability, improved bioavailability, and controlled drug release kinetics. This translates into smaller drug doses necessary to achieve the same therapeutic effect, thus making it possible to minimize side effects and toxicity of pharmacotherapy considerably. In essence, novel DDSs are primarily designed to be patient-friendly, safe, and more effective than conventional systems.^1,9,11^ Particularly promising structures for drug carriers are polymer fibers, which can be fabricated by, e.g., electrospinning (solution electrospinning, melt electrospinning, magnetic-assisted electrospinning, multinozzle electrospinning, nozzleless electrospinning), centrifugal spinning, solution blow spinning, or other nonspinning techniques. Among these methods, solution electrospinning is the most versatile.^13^

High voltage is applied between the grounded collector and the spinneret in the solution electrospinning technique. As a result, the polymer solution is charged, and a Taylor cone is formed at the needle tip. Then, the thin fluid jet emitted from the cone tip is rapidly stretched and thinned, while the solvent evaporates. Solidified fibers are deposited on the collector randomly or in aligned patterns. It allows the production of electrospun fibers with diameters ranging from several micrometers to a few nanometers, and diverse architectures such as classical, core/shell, triaxial, hollow, and Janus, porous structures.^14−16^ Various active substances can be trapped inside fibers or chemically bonded to the fiber surface, e.g., hydrophilic or hydrophobic drugs, antibiotics, DNA, RNA, enzymes, proteins, plant extracts, and nanoparticles. Moreover, the specific properties of fibers can be appropriately tailored to suit their potential biomedical application. Diameter, alignment, porosity, and therefore surface-to-volume ratio, mechanical strength, flexibility, and other physicochemical parameters of fibers can be customized. Fibers for various medical applications are being investigated, such as drug delivery systems, biosensors, three-dimensional (3D) scaffolds for tissue engineering, wound dressings, or implant coatings. For instance, a thin fibrous membrane or thicker nonwoven fiber mat can serve as a coating of implantable device or a dressing, for topical drug administration.^15,17−19^ Apart from various biological and biomedical applications, electrospun fibers can also be utilized across other fields, e.g., water and air filtration, intelligent textiles, information and energy storage, flexible electronics, and food packaging.^19,20^ Therefore, these structures are especially promising for further investigation and development toward large-scale technological applications.

Many commercially available drugs or drug candidates require more adequate carriers, particularly for local administration. Approximately 90% of new candidates for drugs are poorly water-soluble. This significantly impacts their bioavailability and effectiveness.^21^ Furthermore, the drug loading content of nanosized hydrophobic drug delivery systems tends to be very low (below 20%). Consequently, a lot of carrier material must be used, which significantly influences the production cost of DDS and limits its application.^22^ In this context, the drug metronidazole was assessed as an appropriate candidate for conducting our research investigation. Metronidazole (MET) is chemically known as 2-methyl-5-nitroimidazole-1-ethanol, a poorly water-soluble derivative of the compound 5-nitroimidazole. It is an antiprotozoal and antibacterial drug. MET is commonly prescribed for treating many infections, including gingivitis, periodontitis, bacterial vaginosis, rosacea, and numerous postsurgical anaerobic infections, e.g., sepsis, ulcers, and bedsores. Currently, this drug can be administered through three routes: oral (tablets), parenteral (intravenous injection solutions), or topical (gels, creams, and ointments). However, since systemic administration of MET often leads to adverse effects, local administration emerges as a preferable option.^23−26^ Some electrospun fiber compositions have already been investigated and evaluated as suitable carriers of metronidazole.^4,27,28^ These drug delivery systems show promising characteristics. However, none have reached commercial or FDA-approved status or even reached the stage of clinical trials. At best, only in vivo studies were conducted.^29,30^ One of the significant challenges to be addressed is maintaining precise control of the drug release rate, as metronidazole typically requires prolonged administration periods. Preventing the undesired initial burst release of drug molecules is essential for obtaining sustained drug release profiles. Prolonged drug release offers numerous benefits, e.g., the ability to maintain a relatively constant drug concentration in plasma by staying within the therapeutic concentration range between the effective and toxic levels.^31^ Unfortunately, the release kinetics of drug molecules from fibers depends on many factors, such as polymer structure and its properties, degree of drug crystallinity, the amount of drug loading, and interactions between polymer and drug molecules. Therefore, the composition of fibers needs to be carefully adjusted.^32^ There are also other potential strategies to achieve delayed release, such as using a core/shell fiber structure, where the drug is exclusively encapsulated in the fiber’s core. In this case, the required prior degradation of the shell can sometimes help suppress the burst release.^27,28^

Electrospun fibers can be produced from synthetic or natural and hydrophilic or hydrophobic polymers. Biocompatible and biodegradable polymers are particularly well suited for drug delivery and other biomedical applications. They do not elicit an immune response upon contact with native tissue and break down safely within a body.^33^ At present, different types of polymers are being investigated for electrospun metronidazole-loaded fibers, e.g., polycaprolactone (PCL),^27,28,30^ poly(lactic acid) (PDLA, PLLA, PLLA–PDLLA),^4,34^ poly(vinyl alcohol) (PVA),^27^ poly(ethylene oxide) (PEO),^29^ poly(vinylpyrrolidone) (PVP),^32^ Eudragit RL100 (ERL100), and Eudragit S100 (ES100).^27,35^ Inspired by those studies, two different types of polymers were deemed promising for our research: one already commercially used in medical applications (PCL), and the other, significantly less common (P2VP-PS). Polycaprolactone is a semicrystalline, highly hydrophobic synthetic polymer with good flexibility and a low melting point of around 60 °C. It is biodegradable and biocompatible, and has already received FDA approval for use in certain drug delivery systems.^36−38^ On the other hand, poly(2-vinylpyridine-*co*-styrene) (P2VP-PS) is a synthetic block copolymer composed of hydrophilic P2VP and hydrophobic PS segments.^39^ Amphiphilic copolymers also have great potential for various biomedical applications^40−42^ and electrospun filtration membranes.^43^

In this paper, three different compositions of electrospun fibers were investigated, i.e., (i) fibers made of PCL, (ii) fibers made of P2VP-PS, and (iii) core/shell fibers composed of both PCL and P2VP-PS. Each fiber composition was examined as a potential carrier of a pharmaceutical, metronidazole, in varying concentrations. The primary objective of the study was to characterize these novel drug delivery systems in terms of their morphology, structure, and drug release kinetics by employing the following methods: scanning electron microscopy (SEM), X-ray diffraction (XRD), thermogravimetric analysis (TGA), and ultraviolet–visible (UV–vis) spectroscopy. Herein, we aimed to fabricate defect-free electrospun fiber mats with sustained drug release intended for use as a nonwoven dressing for wounds or for the treatment of periodontitis. Overall, this study highlights the promising potential of electrospun fibers loaded with metronidazoles for future medical applications.

## Methods

### Materials

**Figure 1:**
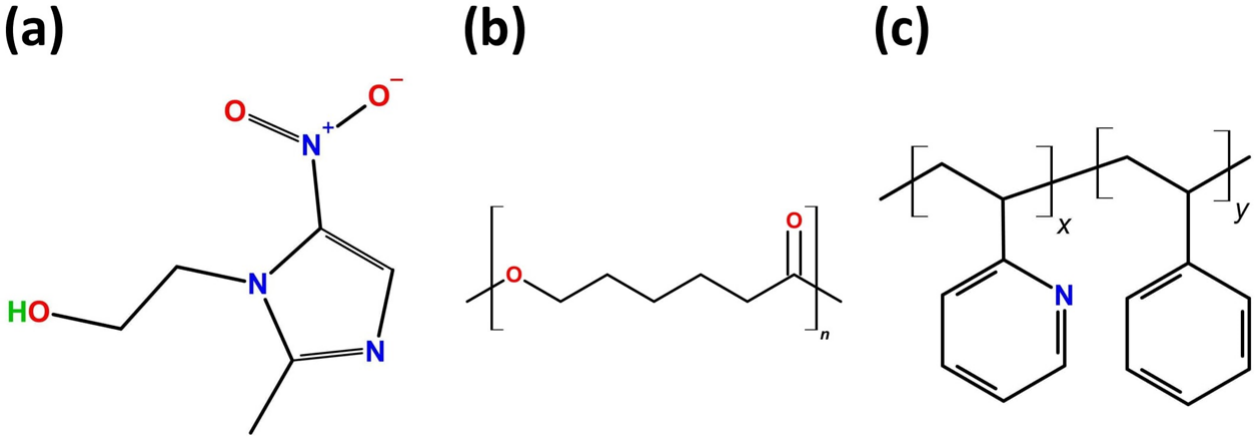
Chemical structures of (a) MET; (b) PCL; and (c) P2VP-PS.

Metronidazole (M3761, CAS: 443–48–1, *M*_w_ = 171.15 g mol^–1^, Figure 1a), PCL (440744, CAS: 2490–41–4, *M*_w_ = 80,000 g mol^–1^, Figure 1b), and P2VP-PS (184608, CAS: 2490–54–9, *M*_w_ = 220,000 g mol^–1^, Figure 1c) were purchased from Sigma-Aldrich. The solvents, chloroform and methanol, were obtained from POCH. All utilized reagents were of analytical grade.

### Electrospinning Process

#### Preparation of Solutions

The initial solutions of 10 wt % PCL and 12 wt % P2VP-PS were prepared by dissolving reagents in a mixture of chloroform and methanol (in a volume ratio 3:1). To obtain fibers with varying drug concentrations, 10, 20, 40, or 50 mg of metronidazole was added per 1 mL of polymer solutions. The mixtures were then stirred overnight to obtain stable homogeneous solutions. All of the formulations were prepared at room temperature.

#### Fabrication of Electrospun Drug-Loaded Fibers

**Figure 2:**
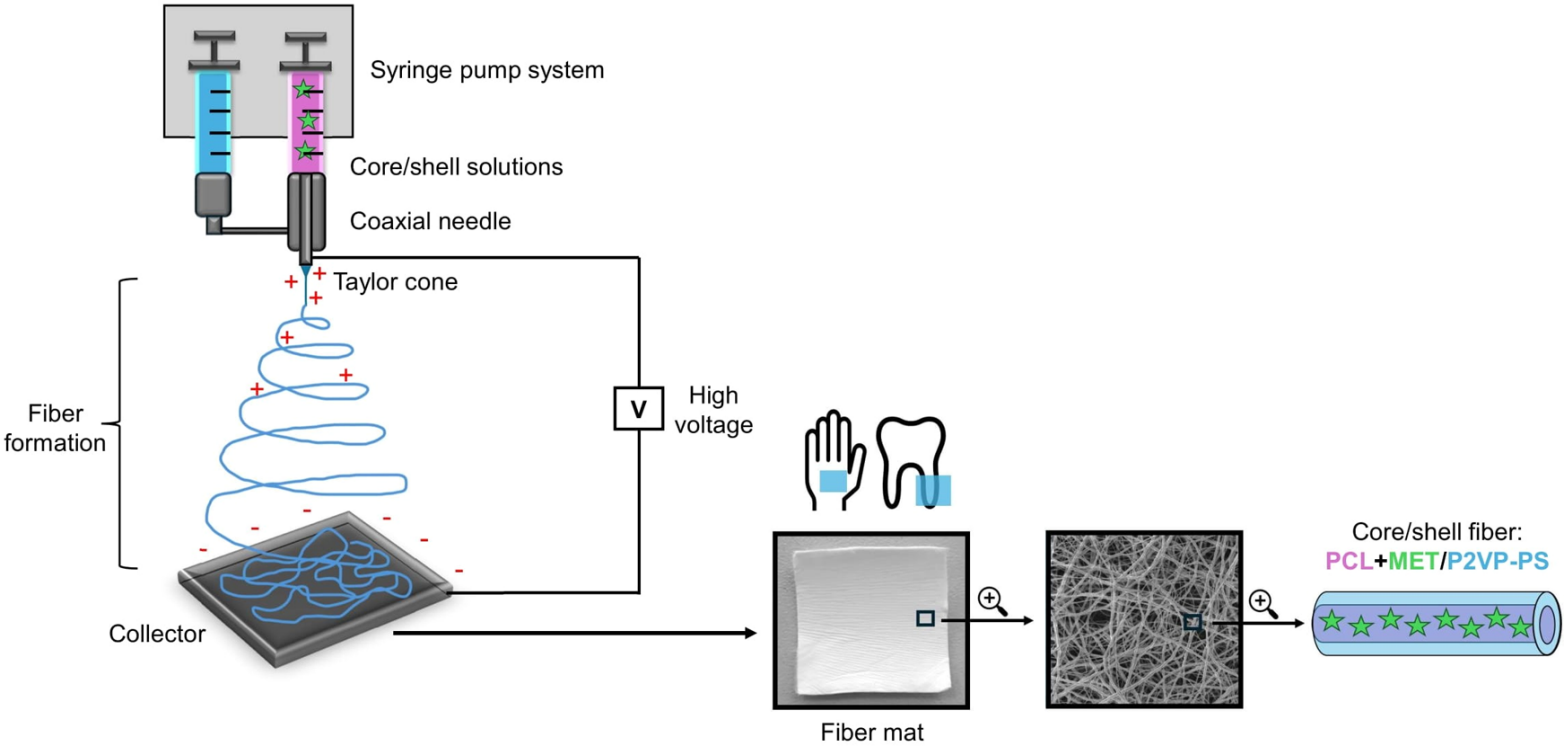
Schematic illustration of a coaxial electrospinning setup.

Fibers with metronidazole were fabricated by using the electrospinning technique (for classical fibers) or the coaxial electrospinning technique (for core/shell fibers) (Figure 2). Drug-free fibers were obtained to serve as a reference. The experimental setup utilized a plate-shaped metal collector to fabricate randomly oriented fibers. A syringe pump system maintained the constant flow rate of polymer solutions (1.5 mL h^–1^ for classical fibers, 1.0 mL h^–1^ for the core solution, and 1.5 mL h^–1^ for the shell solution). The needle tip–collector distance was kept constant at 11 cm. Two needles were used: a hypodermic needle 23G and a coaxial needle 19G/15G. The applied voltage varied based on the solution composition and was optimized through preliminary measurements. The temperature and relative humidity during the electrospinning process were monitored and maintained at 26 ± 2 °C and 55 ± 7%, respectively. Table 1 presents the final compositions of the electrospun fibers along with the corresponding electrospinning parameters. The final samples were in the form of square fiber mats with a side length of approximately 2 cm and a thickness of less than 1 mm.

**Table 1:** Composition and Electrospinning Parameters of the Fabricated Electrospun Fibers

sample code: polymer + drug (*x* mg/mL)	voltage (kV)	solution flow rate (mL h^–1^)	needle gauge and length (cm)	needle tip–collector distance (cm)
PCL	8.5	1.5	23G, 2	11
PCL + MET10	7.0			
PCL + MET20				
PCL + MET40				
PCL + MET50				
P2VP-PS	11.0			
P2VP-PS + MET10	10.5			
P2VP-PS + MET20				
P2VP-PS + MET40				
P2VP-PS + MET50				
core/shell: PCL / P2VP-PS	10.0	1.0/1.5	19G/15G, 1.5	
core/shell: PCL + MET20 / P2VP-PS	13.0			
core/shell: PCL + MET40 / P2VP-PS				

### Characterization of Electrospun Fibers

#### Scanning Electron Microscopy (SEM)

The surface morphology and homogeneity of electrospun fibers were examined using a scanning electron microscope (Tescan, VEGA 3) equipped with a tungsten filament. Measurements were conducted at an acceleration voltage of 1 kV and a working distance of approximately 4 mm. The fiber diameter distributions were obtained using ImageJ software^44^ by measuring diameters at 150 random points on each SEM micrograph.

#### X-ray Diffraction (XRD)

The structure and crystallinity of fibers were determined and monitored over 14 months using an X-ray diffractometer (PANalytical, X’Pert PRO). Measurements were carried out at room temperature, using CuKα radiation (λCuKα_1_ = 1.5406 Å, λCuKα_2_ = 1.5444 Å)^45^ in Bragg–Brentano θ-θ geometry. Samples were scanned from 2 to 40° 2θ angle at a scanning rate of 0.04 or 0.08°/s and a measuring step of 0.033°. X-ray diffraction patterns were analyzed using FullProf software.^46^

#### Thermogravimetric Analysis (TGA)

The drug content in the fibers was quantified using a thermogravimetric analyzer (TA Instruments, TGA 5500). Samples were heated at a rate of 5 °C/min from room temperature to 700 °C under a nitrogen atmosphere using platinum crucibles. The obtained TGA and DTG curves were analyzed by using TRIOS software.

#### UV–Vis Spectroscopy

In vitro drug release profiles were determined by measurements of the concentration of released metronidazole by using a UV–vis spectrophotometer (Ocean Optics, USB2000). Electrospun fiber mats were entirely immersed in deionized water. The geometry of fiber mats was kept constant (squares 2 cm **×** 2 cm), sample weight was in the range of 10–20 mg and water volume varied between 10 and 60 mL depending on the concentration of MET in fiber mats. At specific time intervals over 3 h, the absorbance of a solution containing water and released metronidazole molecules was measured. The percentage of drug released was determined using obtained absorption spectra and calibration curve of metronidazole in water, taking into account the weight of the fiber mat, the volume of water, and the total drug content in the fibers. The experiments were performed at room temperature under static test conditions (no stirring). Drug release profiles and kinetic mathematical models were plotted and fitted using Origin software.

## Results and Discussion

### Morphology and Size Distribution

**Figure 3:**
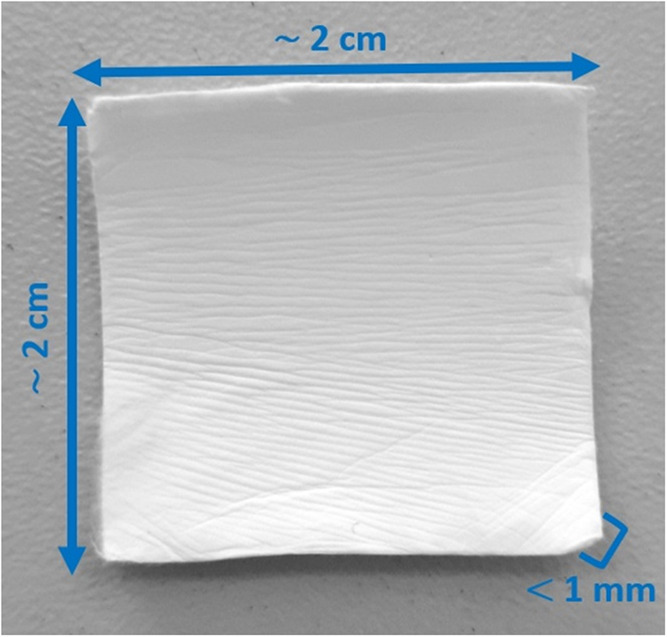
Electrospun fiber mat composed of PCL + MET20.

**Figure 4:**
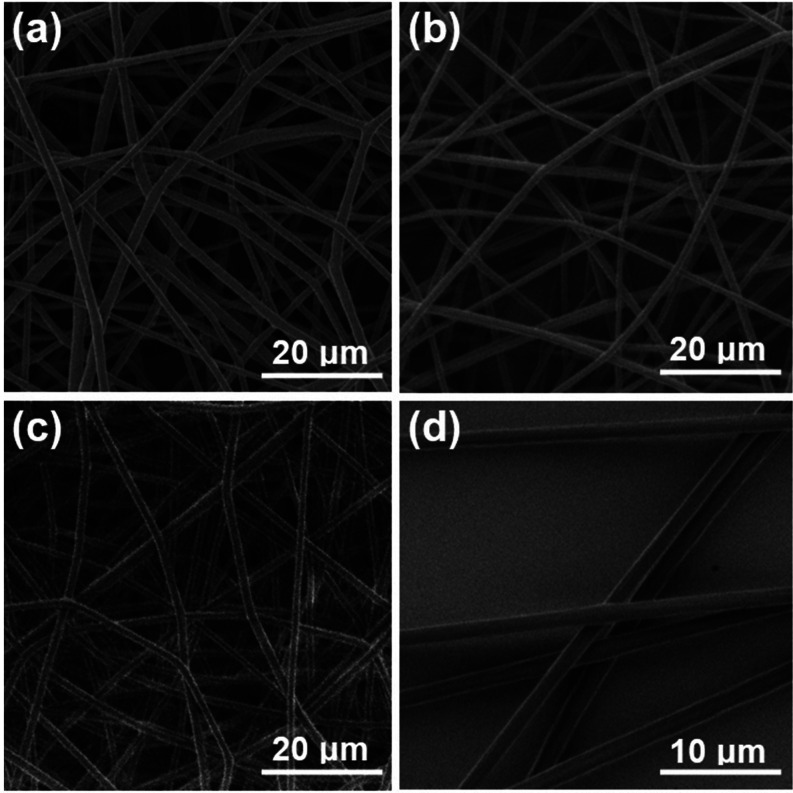
Representative SEM micrographs of electrospun fibers: (a) PCL + MET20; (b) P2VP-PS + MET20; (c) PCL + MET20 / P2VP-PS; and (d) SEM micrograph of P2VP-PS + MET40 fibers at higher magnification.

In the first stage of our study, we optimized electrospinning conditions for different fiber compositions with and without the drug. As a result, long cylindrical randomly oriented fibers were obtained. Since the desired final product is a nonwoven dressing, the electrospinning process lasted about 30 min to obtain an electrospun fiber mat instead of a fibrous membrane. Final fiber mats (Figure 3) were composed of stacked layers of randomly oriented and intertwined fibers. All fabricated fibers were nonporous and free from any significant defects; no beads or aggregations of crystallized drug particles were present (Figure 4). These results indicate that drug molecules were effectively and completely encapsulated within fiber structure and probably homogeneously dispersed. Overall, the surface morphology of all obtained fibers was deemed satisfactory. Regarding only morphology, no significant differences were found between the same polymer fibers with different drug concentrations. However, some differences occurred for various polymers used in fibers (PCL, P2VP-PS, PCL / P2VP-PS). Generally, fibers made of P2VP-PS were more homogeneous and smooth compared to the PCL and core/shell fibers. This indicates that copolymer P2VP-PS is slightly better suited as a base material for electrospun fibers with MET. Fibers made of P2VP-PS exhibit better uniformity and, consequently, better reproducibility.

**Figure 5:**
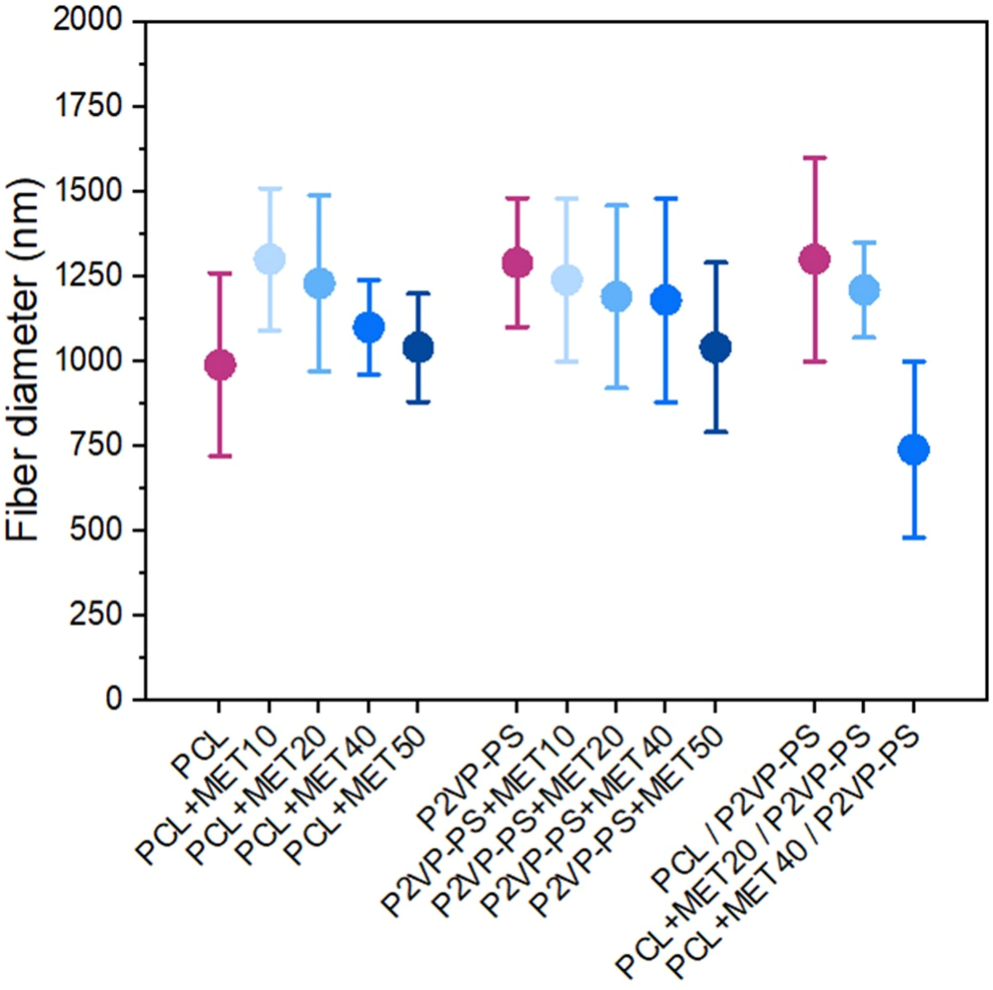
Correlation between electrospun fiber composition and the average fiber diameter.

The average diameter of fibers ranged from about 700 to 1300 nm, depending on the composition (Figure 5). For fibers without the drug, the average diameter was 990 ± 270 nm (for PCL), 1290 ± 190 nm (for P2VP-PS), and 1300 ± 300 nm (for PCL / P2VP-PS). Moreover, there is a clear correlation between MET content in fibers and fiber diameter, present for all three formulations. Namely, a slight decrease in fiber diameter corresponding with an increase of MET concentration in fibers was noted. Since all electrospinning process parameters were fixed, this phenomenon can be probably attributed to the changing electrospinning solution properties (e.g., lower viscosity, higher conductivity) caused by the increase of drug concentration in polymer solution.^14^

### Structural Analysis

Pharmaceuticals can exist in different structural forms: crystalline, semicrystalline, or amorphous. Generally, decreasing drug crystallinity is desired in drug delivery systems since amorphous drugs possess several benefits, such as increased solubility and bioavailability. However, they are thermodynamically unstable and tend to recrystallize over time, making them difficult to manufacture and store properly.^21,26^

**Figure 6:**
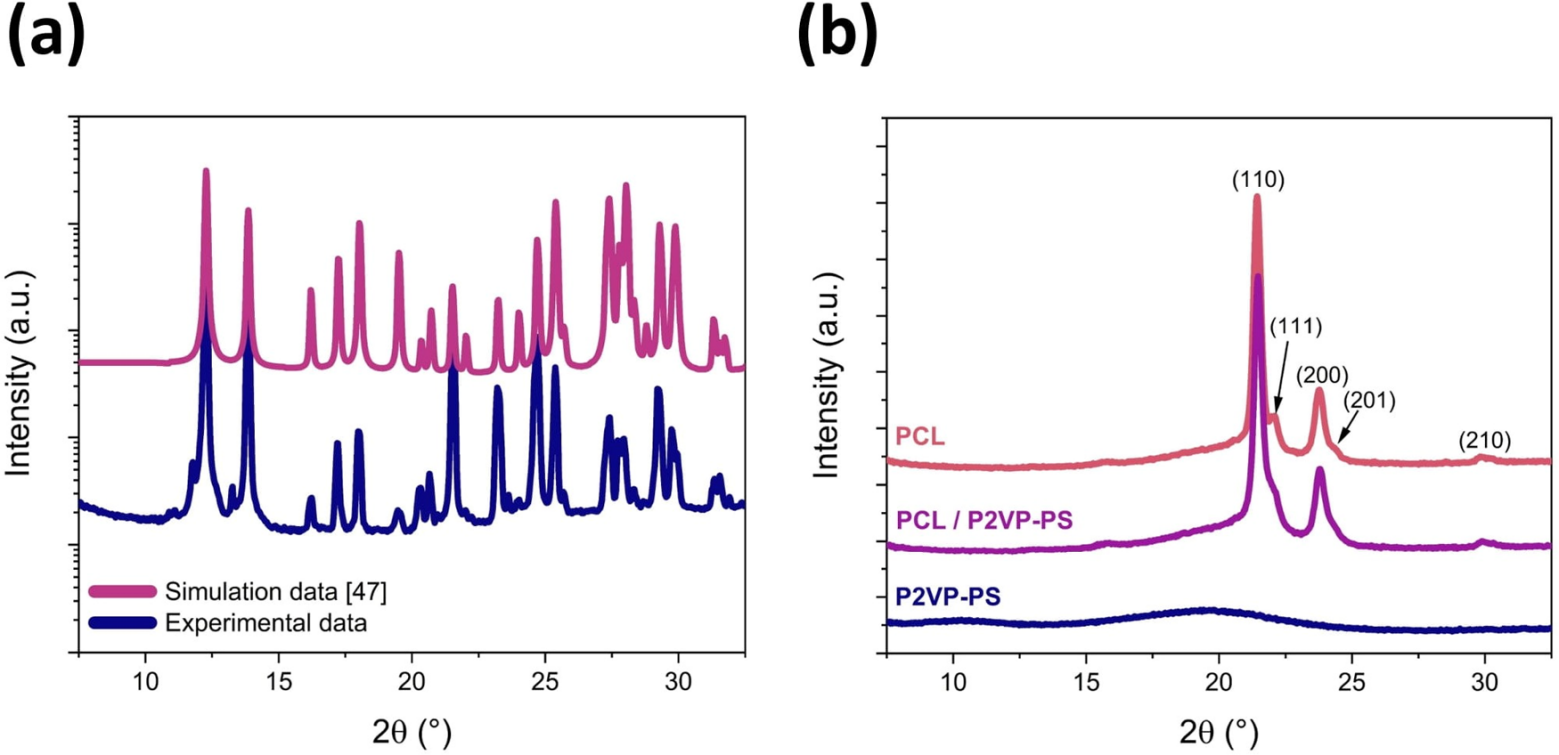
XRD patterns of (a) pure metronidazole and (b) electrospun drugless polymer fibers.

Metronidazole as an API is a highly crystalline material; its structure belongs to the *P*2_1_/*c* space group. The lattice constants of MET are *a* = 7.034 Å, *b* = 8.725 Å, *c* = 12.818 Å, and α = γ = 90°, β = 94.51°.^47^ Its X-ray diffraction pattern is shown in Figure 6a. Several well-defined intense diffraction peaks are present at, e.g., 12.3, 13.9, 21.5, 24.7, 27.4, 27.9 and 29.3°. Moreover, XRD patterns of drug-free electrospun fibers are presented in Figure 6b. Both PCL and PCL / P2VP-PS fibers can be classified as semicrystalline materials (the orthorhombic unit cell: *a* = 7.47 Å, *b* = 4.98 Å, *c* = 17.05 Å and α = β = γ = 90°, space group *P*2_1_2_1_2_1_^48^) with three main peaks at 21.4, 23.8 and 29.9°. Whereas P2VP-PS fibers are amorphous, with the diffraction halo centered at 19.2°.

**Figure 7:**
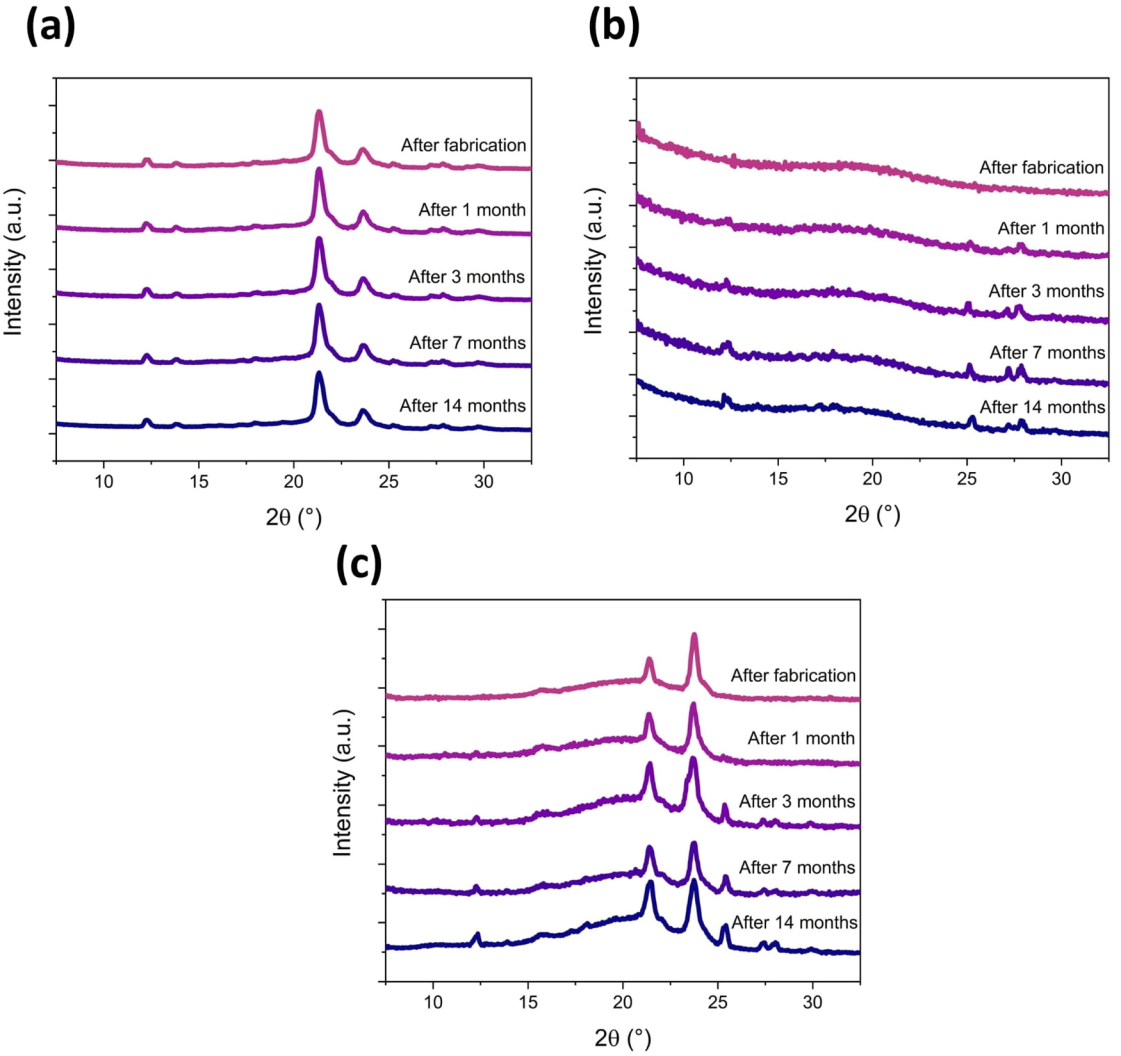
Changes in XRD patterns of electrospun fibers over a storage period for selected samples: (a) PCL + MET20; (b) P2VP-PS + MET20; (c) PCL + MET20 / P2VP-PS.

XRD analysis was carried out for three representative samples: PCL + MET20, P2VP-PS + MET20, and PCL + MET20 / P2VP-PS in specific time intervals over a 14-month storage period in the dark at room temperature. The aim of this study was to estimate the amorphous/crystalline form of MET in fibers and its recrystallization rate over time. For PCL + MET20 fibers (Figure 7a), some peaks related to the presence of metronidazole can be observed, e.g., 12.3, 13.8, 25.2, 27.2, and 27.9°. However, the intensity of these peaks is noticeably tiny, which suggests the decrease in crystallinity of metronidazole in fibers compared to the pure metronidazole. There are no visible changes in the intensity and number of peaks over time, indicating the structural stability of this drug-carrier system. Conversely, for P2VP-PS + MET20 fibers (Figure 7b), no distinct peaks corresponding to metronidazole are visible immediately after fiber fabrication. Therefore, it can be assumed that the drug in fibers is amorphous, or at least mainly amorphous. After one month, some peaks from MET became slightly noticeable (12.3, 25.1, 27.2, 27.9°). And in the following months, the slow recrystallization of MET is clearly detectable. A similar situation occurs for the core/shell PCL + MET20 / P2VP-PS fibers (Figure 7c). Up to one month, no peaks of crystalline MET were detected, suggesting the amorphization of metronidazole in fibers. The recrystallization of MET slowly occurred during storage, observed by the increasing intensity of peaks at 12.3, 25.4, 27.4, and 28.0°. There might be various reasons for the decrease in drug crystallinity during electrospinning. During this process, intermolecular interactions between polymer chains and drug molecules undoubtedly play a crucial role in the observed amorphization.^26^

**Figure 8:**
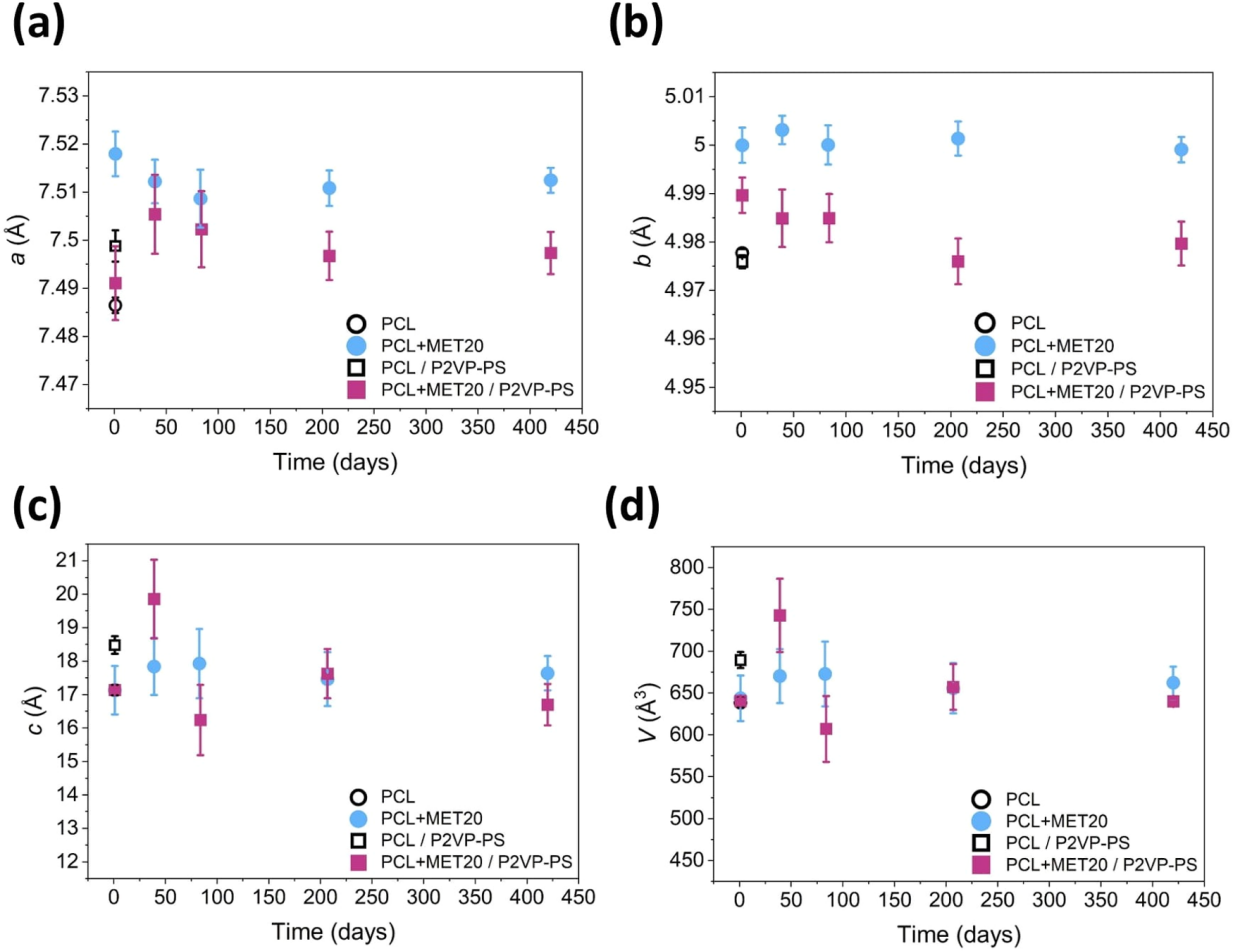
Changes in lattice constants: (a) parameter *a*, (b) parameter *b*, (c) parameter *c*, and (d) volume of unit cell; for selected samples of electrospun fibers, over a storage period.

Additionally, the structural stability of the obtained drug-carrier systems was verified by monitoring changes in lattice parameters over time for two representative samples: PCL + MET20 and PCL + MET20 / P2VP-PS (Figure 8). Overall, no significant changes were observed for *a*, *b*, *c*, and *V* values, indicating the structural stability of fabricated electrospun fibers with drug.

### Drug Loading Studies

**Figure 9:**
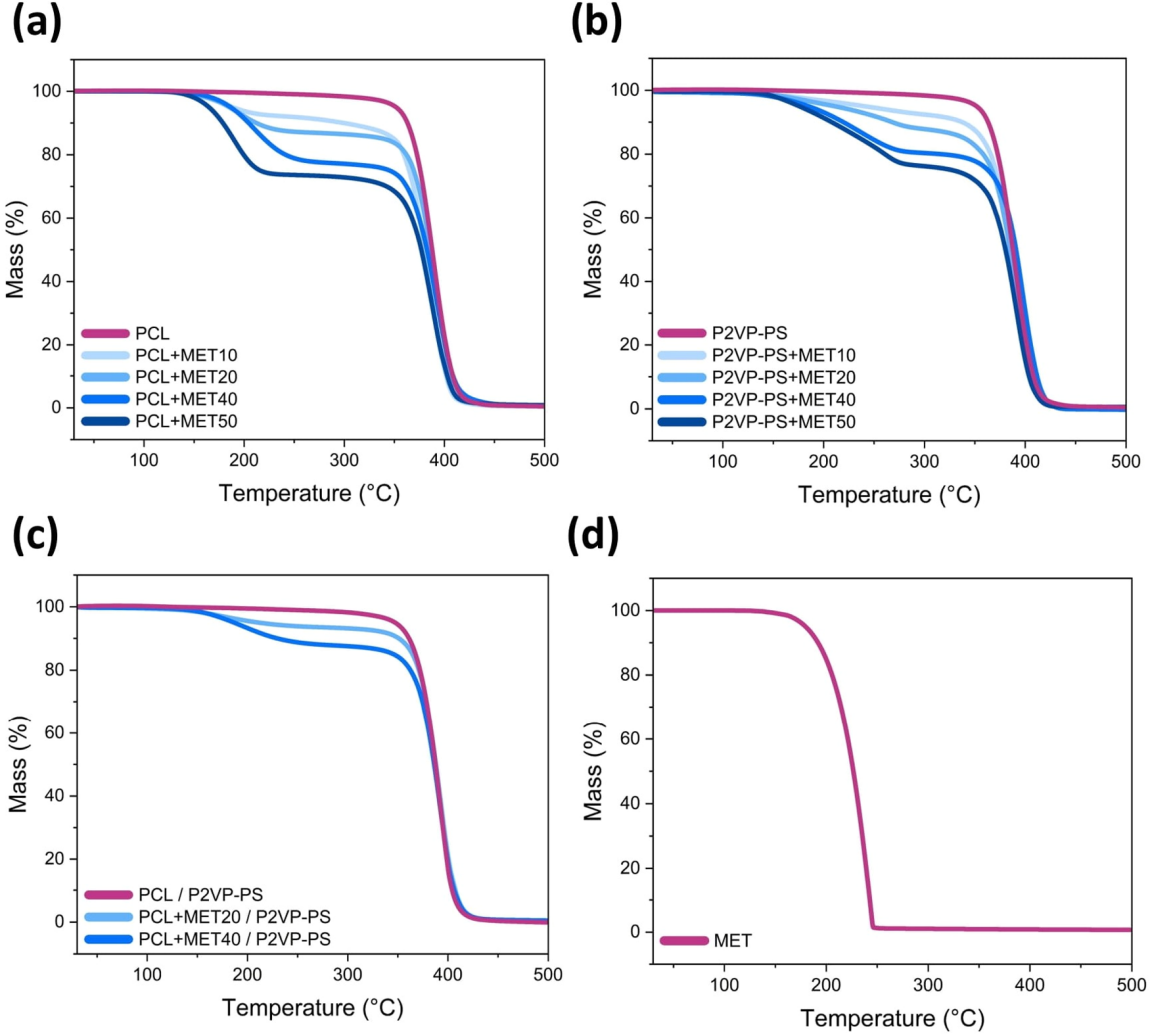
TGA curves of electrospun fibers composed of (a) PCL; (b) P2VP-PS; and (c) PCL and P2VP-PS. (d) TGA curve of pure metronidazole powder.

Thermogravimetric analysis was used to determine the actual weight percentage of the drug within fibers and therefore to obtain drug loading content (DLC) values. Thermal decomposition of polymers and the drug is a one-step process and occurs in different temperature ranges. Pure metronidazole powder degrades between ca. 211 and 246 °C, whereas polymer fibers degrade in a higher temperature range of about 366–409 °C (for PCL), 379–412 °C (for P2VP-PS), and 369–409 °C (for PCL / P2VP-PS) (Figure 9). Consequently, polymer electrospun fibers with metronidazole degrade in two main steps. The first mass loss (below 300 °C) corresponds to the thermal degradation of metronidazole. The second mass loss (above 300 °C) relates to the degradation of polymers: PCL and/or P2VP-PS. Residue is negligible (less than 1%). The solvents (methanol and chloroform) used for electrospinning solutions were not detected, indicating their total evaporation during fiber formation. No significant mass loss (less than 0.1%) occurred in the temperature range of 50–80 °C encompassing boiling points of methanol (64.5 °C) and chloroform (61.1 °C).^49,50^ Additionally, the shape of TGA curves (for the first mass loss) varies slightly between types of polymers used in fibers. If P2VP-PS is present, the curve slope is less steep, indicating slower weight loss associated with the thermal decomposition of metronidazole. Moreover, with increasing MET content, small changes in the onset and offset temperature values for each decomposition stage were noticed.

The drug loading content for electrospun fibers was determined using the following equation:^22,51^
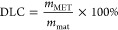
1where *m*_MET_ is the mass of MET present in the fiber mat and *m*_mat_ is the total mass of the fiber mat.

**Figure 10:**
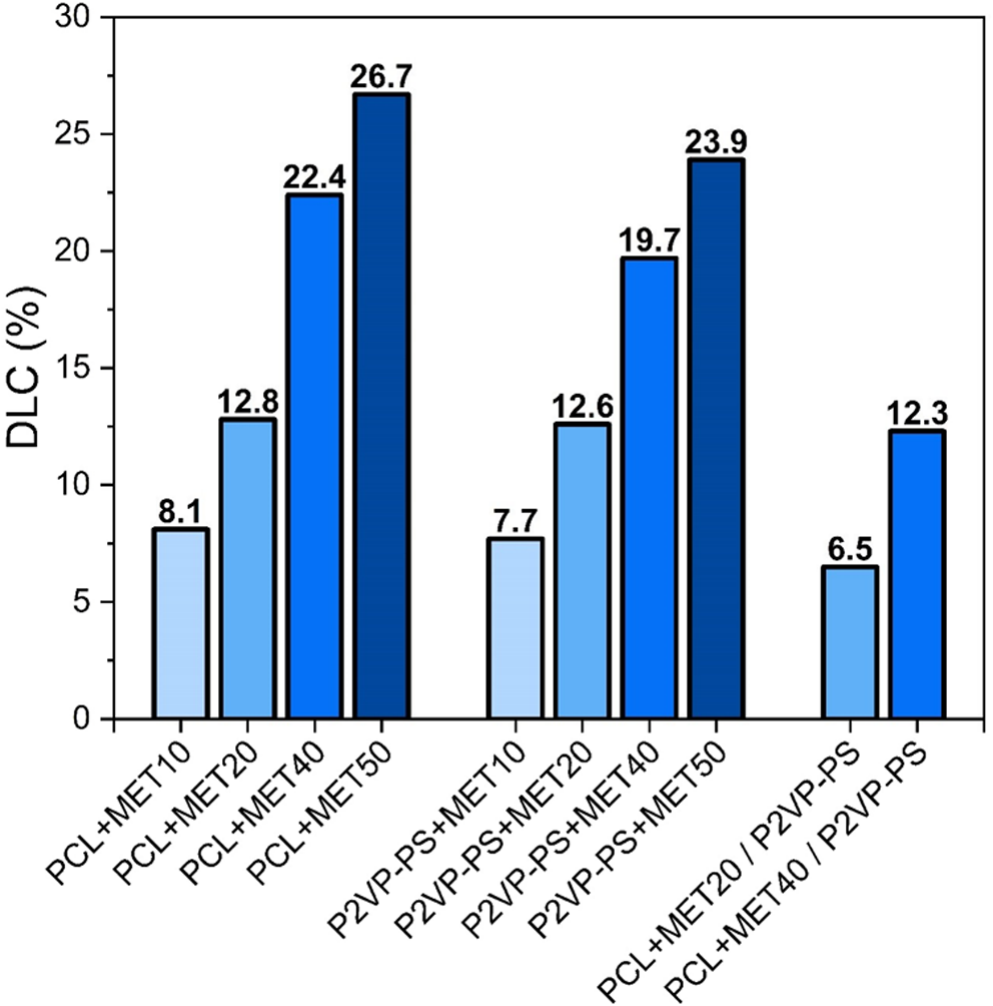
Drug loading content for different compositions of electrospun fibers.

The obtained DLC values are summarized in Figure 10. As expected, a higher concentration of metronidazole in the initial electrospun solution translated into a higher DLC value. The highest obtained values for PCL + MET and P2VP-PS + MET fibers are 26.7 and 23.9 wt %, respectively. Furthermore, calculated values for PCL + MET fibers tend to be slightly higher than those for the corresponding P2VP-PS + MET fibers, which suggest that PCL fibers possess slightly better loading capacity. On the other hand, DLC values for core/shell fibers are 6.5 and 12.3 wt %. There are approximately 50% lower values than those for the corresponding PCL + MET20 and PCL + MET40 classical fibers. This is due to the presence of the shell composed of P2VP-PS without any added drug. Furthermore, these numbers indicate that the designed coaxial electrospinning process is reproducible, enabling the production of core/shell fibers with approximately consistent shell thickness.

### In Vitro Drug Release Studies

**Figure 11:**
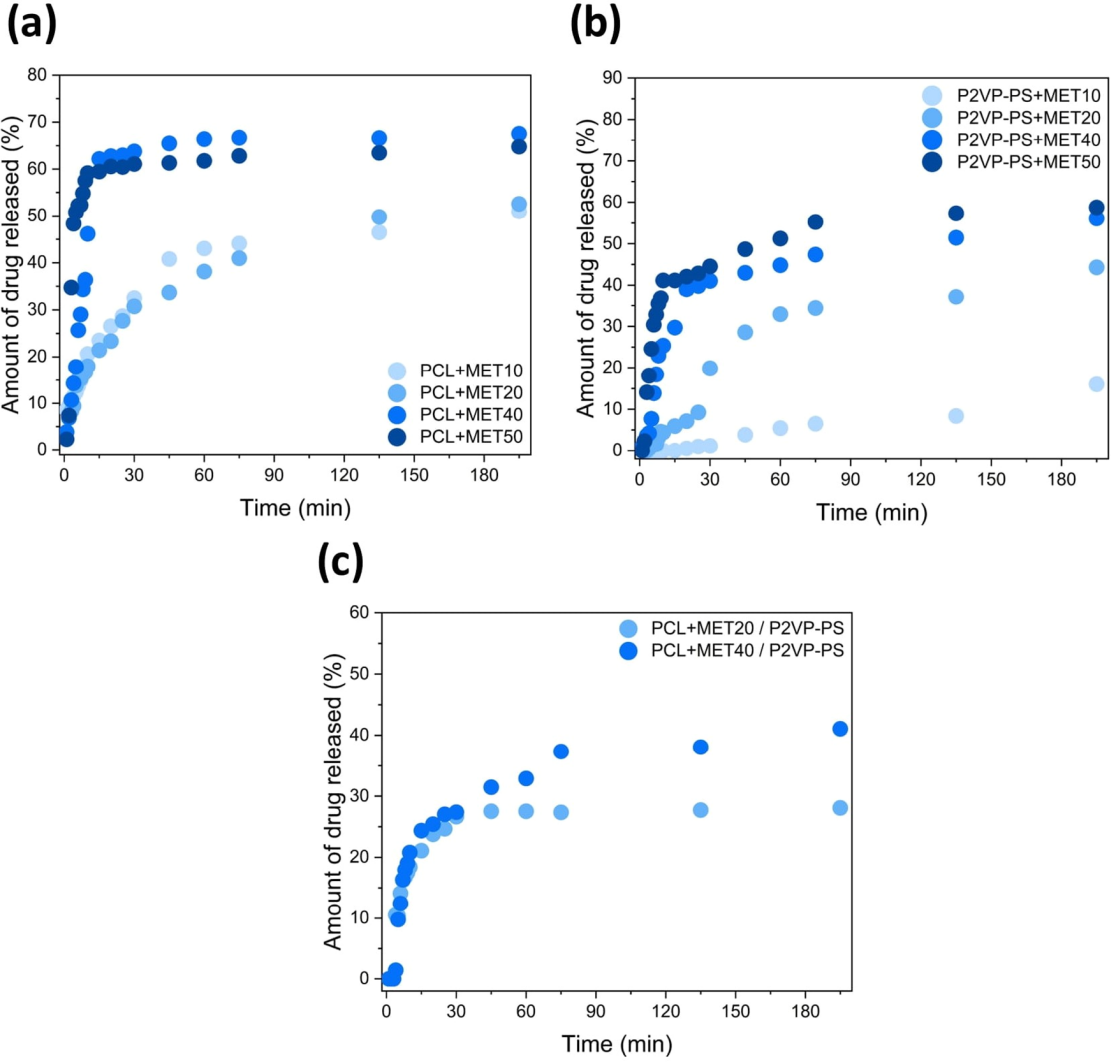
In vitro release profiles of metronidazole from electrospun fiber mats composed of (a) PCL + MET; (b) P2VP-PS + MET; and (c) PCL + MET / P2VP-PS.

Drug release profiles provide helpful information about the mechanism, rate, and extent to which the drug is released from the carrier over a specific time interval. Obtained results are crucial to verifying whether the drug-carrier system suits its potential medical application.^52^Figure 11 shows obtained in vitro drug release profiles plotted as a percent of metronidazole released as a function of time. First and foremost, graphs show a strong correlation between the drug release rate and the drug concentration in fibers. A higher concentration of metronidazole in fibers corresponds to its faster release, which is especially clear for all P2VP-PS + MET and PCL + MET / P2VP-PS formulations. These results are in line with the general trend that higher drug loading causes faster drug release.^32^ Moreover, the burst effect can be observed for fibers with the highest concentration of MET. For instance, for PCL + MET50 fibers, approximately 50% of MET was released in the first 5 min of a study. Conversely, for lower MET concentrations (e.g., PCL + MET20), 50% of MET was released noticeably later (after 3 h). Thus, for lower MET concentrations in fibers, the release is considerably more controlled. Interestingly, for P2VP-PS + MET10, the almost linear drug release was observed, indicating possible zero-order kinetics behavior. In the case of core/shell fibers, the achieved inhibition of the burst effect is evident. These findings confirm the hypothesis that core/shell fiber structure can effectively suppress undesirable drug burst effect.^27^

Drug release kinetic models are commonly used to mathematically describe the process of drug release from carriers. There are four main drug release models: zero-order kinetics (eq 2), first-order kinetics (eq 3), Higuchi kinetics (eq 4), and Korsmeyer–Peppas kinetics (eq 5):
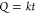
2
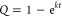
3
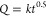
4
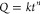
5where *Q* is the fraction of released drug at a specific time *t*, *k* is the release constant, and *n* is the release exponent.^27,53^ The fitting of the Korsmeyer–Peppas model yielded the highest adjusted *R*^2^ values for all analyzed samples, indicating the best suitability to our drug-carrier systems. The fitting parameters for this model are presented in Table 2. Higher values of parameter *k* correspond with the faster release rate of the drug from the carrier.^52^ The *k* values obtained for most samples are relatively low, except for PCL + MET40, PCL + MET50, and P2VP-PS + MET50. For these electrospun fiber compositions, the values are noticeably higher (*k* > 0.1), clearly indicating the presence of burst effects. These numbers are in obvious accordance with previous visual assessments of the drug release profiles. On the other hand, the value of parameter *n* implies which release mechanism is present in the drug-carrier system. Overall, since fibers classify as thin cylindrical carriers, the relationship is as follows: *n* < 0.45 (Fickian diffusion mechanism), 0.45 < *n* < 0.89 (non-Fickian transport), *n* = 0.89 (Case II transport), *n* > 0.89 (super Case II transport).^52^ For all samples, except P2VP-PS + MET10 and P2VP-PS + MET20, calculated *n* values are less than 0.45. These results suggest that the Fickian diffusion mechanism occurs for almost all investigated fiber compositions. Only for P2VP-PS + MET20 fibers, parameter *n* = 0.651 indicates anomalous (non-Fickian) diffusion mechanism, and for P2VP-PS + MET10 fibers, parameter *n* = 1.144 suggests super Case II transport present.

**Table 2:** Drug Release Kinetic Model (Korsmeyer–Peppas) Fitting Parameters

sample code	*k*	*n*	adjusted *R*^*2*^
PCL + MET10	0.0882 ± 0.0080	0.353 ± 0.022	0.9492
PCL + MET20	0.0724 ± 0.0050	0.391 ± 0.016	0.9766
PCL + MET40	0.191 ± 0.036	0.284 ± 0.048	0.7409
PCL + MET50	0.336 ± 0.046	0.155 ± 0.039	0.6420
P2VP-PS + MET10	0.00037 ± 0.00015	1.144 ± 0.080	0.9649
P2VP-PS + MET20	0.0163 ± 0.0053	0.651 ± 0.070	0.8903
P2VP-PS + MET40	0.094 ± 0.017	0.365 ± 0.044	0.8448
P2VP-PS + MET50	0.169 ± 0.023	0.264 ± 0.036	0.8157
PCL + MET20 / P2VP-PS	0.090 ± 0.015	0.254 ± 0.042	0.7721
PCL + MET40 / P2VP-PS	0.071 ± 0.013	0.362 ± 0.045	0.8350

Furthermore, the visual assessment of fiber mats was done during and immediately after the drug release process to observe their behavior in an aqueous environment. All mats wholly maintained their structural integrity, and no disintegration or dissolving of matrices was noticed. For PCL + MET fiber mats, the swelling was not observed, and for PCL + MET / P2VP-PS fiber mats, the swelling was slightly noticeable. On the other hand, for P2VP-PS + MET mats especially with lower concentrations of drug, the swelling was clearly perceivable which corresponds with anomalous release mechanism present for these samples. Lastly, the ability to absorb water was estimated for fiber mats without drug by weighting mats before and after 3 h of immersion in water. The swelling ratio (SR) was then calculated using the following equation:^54,55^
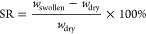
6where *w*_swollen_ is the weight of the swollen fiber mat and *w*_dry_ is the weight of the dry fiber mat.

PCL fiber mats exhibit low water absorption ability (SR = 53%), while PCL / P2VP-PS and P2VP-PS fiber mats possess higher absorption capacity (SR = 370% and SR = 560%, respectively). The results are in good accordance with the degree of hydrophilicity of polymers used, namely, the high hydrophobicity of PCL and amphiphilicity of P2VP-PS.^38,39^ The absorption ability values suggest the most suitable target of proposed mats/dressings—wounds with mild, moderate, or heavy exudate production.^56,57^

## Conclusions

Altogether, this work highlights the promising potential of utilizing electrospun polymer fibers as drug delivery systems. Electrospun fibers composed of different polymers (PCL, P2VP-PS), exhibiting diverse architectures (classical, core/shell), and encapsulating varying concentrations of metronidazole (0, 10, 20, 40, 50 mg/mL) were successfully fabricated and characterized. The SEM micrographs showed smooth and defect-free fibers with average diameters ranging from about 700 to 1300 nm, depending on the fiber composition. The amount of encapsulated metronidazole did not noticeably influence the fibers’ surface morphology. However, a slight decrease in fiber diameter corresponding to an increase of MET content in fibers was observed. For P2VP-PS + MET and core/shell formulations, amorphization of metronidazole in the fibers was observed. For PCL + MET fibers, only a decrease in crystallinity of metronidazole was recorded. The slow recrystallization of drug began approximately one month after fibers fabrication. Moreover, no significant changes in lattice parameters were observed during a 14-month storage period. Furthermore, the drug loading content values for fibers were found to be between ca. 7 and 27 wt % of MET, depending on the composition. The in vitro drug release kinetics were also investigated to evaluate the controllability of the release. The values obtained from fitting the Korsmeyer–Peppas model indicate that the mechanism of drug release is based on the Fickian diffusion for all compositions except P2VP-PS fibers with lower concentrations of metronidazole. For these formulations, the non-Fickian or super Case II transport is present, and thus the most sustained release is achieved. The drug burst effect is present only for fibers with the highest concentrations of MET and can be effectively suppressed by using the core/shell fiber architecture. Fiber mats made of PCL exhibit 7 and 11 times lower water absorption capacity compared to P2VP-PS and core/shell fiber mats, respectively. All things considered, our findings indicate that electrospun fibers made of P2VP-PS + MET possess the most favorable characteristics. Therefore, this makes them worthwhile for further more complex investigation.
